# The use of memantine for prevention of Alzheimer's disease: Pilot feasibility study rationale and protocol

**DOI:** 10.1016/j.conctc.2026.101644

**Published:** 2026-05-04

**Authors:** Anna M. Arp, George S. Bloom, Anelyssa D'Abreu, Amy Fansler, Kimberlee Meegan, Sarah Ratcliffe, Jaideep Kapur, Carol Manning

**Affiliations:** a Brain Institute, University of Virginia, United States; b Department of Biology, College and Graduate School of Arts & Sciences, University of Virginia, United States; c Department of Neuroscience, School of Medicine, University of Virginia, United States; d Department of Cell Biology, School of Medicine, University of Virginia, United States; e Department of Neurology, School of Medicine, University of Virginia, United States; f Public Health Sciences, School of Medicine, University of Virginia, United States

**Keywords:** Alzheimer's disease, Prevention

## Abstract

**Background:**

Alzheimer's disease (AD) affects over 6 million older adults in the Untiled States. Evidence suggests the neuropathology leading to the disorder begins decades earlier, calling for a preventative treatment that can be administered to at risk individuals. Memantine hydrochloride, an NMDA receptor antagonist, is a possible candidate for prophylactic treatment by diminishing excessive NMDA receptor activity.

**Methods:**

This is a 2-year, double-blind, randomized, placebo-controlled trial of memantine hydrochloride (1:1 randomization allocation using randomly permutated blocks of unequal size). Participants are APOε4 carriers slightly under the average age of AD symptom onset (50-65 years of age) with a positive family history of a first degree relative with AD. Amyloid PET scans are performed pre and post treatment. Cognitive assessments and physical and neurological examinations are completed at regular intervals throughout the feasibility trial.

**Discussion:**

This study will assess the feasibility of the use of memantine hydrochloride for prevention of AD. The primary aim is to determine feasibility of participants who a) enrolled among those found eligible, and b) completed the study among those randomized to a study arm. Exploratory aims include examination of cognitive and safety assessments. Although not powered to determine efficacy, the study will provide direction on design elements needed for a Phase III clinical trial. No formal hypotheses are included in this feasibility trial.

**Trial registration:**

Clinical Trials NCT05063851.

## 1 Introduction

As the US population ages, the prevalence of dementia is increasing with Alzheimer's Disease (AD) accounting for 60% to 80% of all dementias [1,2]. The dysfunction of synapses and eventual neuronal death leading to AD pathology starts up to 20 or more years before clinical symptoms appear [2]. There is a large body of evidence to suggest a role for NMDA receptors in the neuropathology of AD [[3], [4], [5], [6]]. Choi [7] found that excessive glutamate in synaptic and extra-synaptic space can lead to chronic over-activation of NMDA receptors, which leads to activation of neurodegeneration pathways [7]. Additionally, there is often excessive production or accumulation of toxic forms of amyloid β (Aβ) in AD [8]. Interestingly, several studies have found that NMDA receptor function has been modulated by Aβ [3,9] and tau [6]. As such, addressing the AD epidemic is likely to require preventive therapy beginning many years before symptom-onset in at-risk individuals.

Preclinical findings suggest that memantine hydrochloride, an NMDA receptor antagonist, has previously undetected disease-modifying properties by blocking adverse downstream effects of NMDA receptor overactivity, such as ectopic neuronal cell cycle re-entry (CCR), a prelude to as much as 90% of neuron death in AD [10,11]. It follows naturally that blocking this ectopic neuronal CCR might prevent or delay AD symptom onset and slow the rate of symptom progression. Any drug that could accomplish such a goal would qualify as a disease-modifying therapy for AD. Based on this research, we developed a protocol to test the feasibility of using memantine hydrochloride for the prevention of AD in a population of asymptomatic individuals who are at increased risk for AD based on APOε4 carrier status and positive family history of AD.

Memantine hydrochloride is one of few FDA-approved drugs for AD treatment, and it targets damaged NMDA receptors while minimally affecting normally functioning receptors. However, FDA approval of memantine in 2003 to treat AD was based on clinical trials of patients who were already symptomatic, and approval was confined to moderate to severe cases [11]. Until now, clinical trials of memantine use in AD have focused on treatment, not prevention. In light of the evidence that memantine blocks ectopic neuronal cell cycle re-entry, a key early step in AD pathogenesis [10,12], repurposing memantine as a potential AD prophylactic is amply warranted.

### 1.1 Research aim

The primary objective of this study is to determine the feasibility of the use of memantine hydrochloride as a prophylactic treatment in pre-symptomatic individuals at high risk of dementia caused by AD. The secondary objective is to estimate the design elements needed for a Phase III clinical trial. No formal hypotheses are included in this feasibility trial.

## 2 Methods

### 2.1 Study design

This is a 2-year double-blind, randomized, placebo-controlled trial of memantine as a prophylactic in asymptomatic, at-risk participants (APOε4 positive, age 50-65 years, with a first degree relative with AD). The study includes randomization and blinding in the design to establish feasibility for a later phase study. Long-term follow-up and secondary outcome measure are also included to provide insight into the design elements needed for a larger efficacy study.

**Study Intervention.** Memantine hydrochloride is an orally active NMDA receptor antagonist indicated for the treatment of moderate to severe dementia of the Alzheimer's type [11]. Memantine hydrochloride is available for oral administration as capsule-shaped, film-coated tablets containing 5 mg or 10 mg of memantine hydrochloride [13]. Memantine hydrocholoride dosage for this study is per product labeling for currently approved indication of treatment for moderate to severe dementia of the Alzheimer's type [13]. This dose is known to be safe and effective at modestly and temporarily decreasing symptoms in the on-label population [14]. The sample size in the study precludes a study of variable doses.

The study drug is acquired by, compounded, and distributed by the University of Virginia (UVA) Investigational Drug Services (IDS). To allow for blinding, IDS encapsulates and backfills both memantine hydrochloride and placebo to ensure capsules appear identical. Importantly, participants are instructed that the capsules must remain intact and unopened, which deviates from the package insert.

**Participant recruitment and enrollment.** Participants are recruited primarily from the Virginia Alzheimer's Disease Center Participant Registry and from providers in the multidisciplinary Memory and Aging Care Clinic (MACC) during appointments for the person living with dementia (PLWD). Participants are also recruited directly from the community using advertisements. The Virginia Alzheimer's Disease Center (VADC) Participant Registry stores contact information for individuals with AD and related disorders (ADRD), caregivers, and healthy adults to facilitate recruitment into UVA ADRD research projects. Notably, many of the healthy adult registrants have a family history of cognitive impairment or other risk factors for AD. Potential participants are screened using the criteria in Table 1. Participants are required to enroll with a study participant who can be an informant for cognitive staging.

**Table 1:** Participant inclusion/exclusion criteria.

*Inclusion criteria*	*Exclusion criteria*
Age ≥50 and ≤ 65 at time of consent	Current clinical condition or medication that raises the pH of urine
Positive family history for dementia (minimum 1 first degree relative)	Sever renal or hepatic impairment
Otherwise healthy for their age group or medically stable with or without medication based on baseline screening procedures	Abnormality, degenerative disorder, or current neurological disease (other than preclinical AD) that would cause possible cognitive defect, neurocognitive decline, or difficulty interpreting new neurological symptoms
Basic spoken and written English skills	Contradictions for MRI or PET imaging
Previously know or documented heterozygote or homozygote APOε4 allele	Clinically significant and active, pulmonary, gastrointestinal, renal, hepatic, endocrine, cardiovascular, or psychiatric disorder or disease
MoCA score ≥26	Ongoing cancer treatment
Creatine clearance estimate ≥30 mL/min	Use of an investigational medical devise 3 months prior to starting the study or current participation in an intervention with an investigational drug component.
Has a reliable co-participant able to provide information about the participant	Major surgery with general anesthesia withing 8 weeks before screening
	Requiring AChE inhibitor or acetazolamide, methazolamide, amantadine, ketamine, or dextromethorphan.

**Randomization procedure.** Participants are randomized to either memantine hydrochloride or placebo (hereafter referred to as study drug). The randomization scheme is generated by the study biostatistician using the R statistical package in a 1:1 allocation using randomly permutated blocks of unequal block size. The randomization scheme is provided directly to the UVA IDS to allow for blinding.

**Schedule of Assessments** The privacy rights of the participants have been observed and informed consent was obtained for experimentation with human subjects. The trial is registered on https://clinicaltrials.gov/(Trial Registration: NCT05063851) and has received approval from the University of Virginia Institutional Review Board for Health Sciences Research (HSR200202) with a determination that the radiation exposure is minimal and consistent with clinical research norms. Approval from the UVA IRB to conduct the study was granted on July 2, 2021. The study opened to recruitment the same day and is currently open. Data collection is estimated to be completed by December of 2025. Funding for this study is provided by the Stanley E. Fulton Family Foundation.

Screening visit: The screening visit takes place within 30 days of the Baseline Visit (day 0) and includes collecting a Montreal Cognitive Assessment (MoCA), physical exam, ECG, urinalysis, blood chemistry sample, blood APOε4 testing, and an MRI without contrast. An amyloid PET scan is performed after eligibility is confirmed and before Week 8. The PET scan is not read until the participant completes the second, post-intervention PET scan.

Baseline visit: Participants are randomized to memantine hydrochloride or placebo (study drug) and provided with 5 mg capsules of the blinded study drug. During the baseline visit participants are educated on the titration schedule and drug accountability, blood is collected for specimen banking, and a battery of neurocognitive assessments are administered (Repeatable Battery for the Assessment of Neuropsychological Status (RBANS), Alzheimer's disease Cooperative Study - Activities of Daily Living Inventory (ADCS-ADL), Alzheimer's disease Cooperative Study - Activities of Daily Living Prevention Instrument (ADCS-ADL-PI), Clinical Dementia Rating Scale (CDR) Scale – Subject and Informant/Study Partner, and Center for Epidemiologic Studies Depression Scale (CES-D)).

Up-titration visits: Participants return to the study site at weeks 2, 3 and 4 to increase titration of study drug (10 mg, 15 mg, and 20 mg respectively).

Treatment phase visits: From Week 12 to Week 96 participants return to the study site every 12 weeks to receive additional doses of study drug. A chemistry panel and urinalysis are performed at weeks 12 (chemistry panel only), 48, 72, and 96. Every 24 weeks (24, 48, 72, and 96) the neurocognitive battery is repeated and at every 48 weeks (48 and 96) a physical exam and vital signs are documented.

Down-titration visits: Down-titration occurs at weeks 96, 97, and 98 or if the participant discontinues treatment early based on the clinical judgement of the study investigator. An MRI without contrast and an amyloid PET scan are performed after the week 96 visit.

Safety Follow-up visit: Participants return to the study site at week 101 or within 14-28 days after the final dose of study drug for final safety assessment.

Study partner visits: Study partners complete the ADCS-ADL and ADCS-ADL-PI scales and participate in the CDR interview at baseline and weeks 24, 48, 72, and 96.

### 2.2 Analytic plan

**Analytic overview.** As a feasibility study, no formal hypothesis testing is planned. Outcomes are preliminary and exploratory and provide estimates needed to design a Phase III efficacy study (see Table 2.). The primary aim is to examine feasibility based on the proportion of participants who a) enrolled among those found eligible, and b) completed the study among those randomized to an arm. Exploratory aims include examination of cognitive and safety assessments between intervention and control groups. Although the trial is not powered to determine efficacy, it will provide direction for design of a larger Phase III efficacy trial.

**Table 2:** Study measures.

Outcome	Measure	Measure Detail
Primary	Enrollment and lost to follow-up	Percentages of eligible participants to enroll and randomized participants to complete the trial
Secondary - Efficacy	Repeatable Battery for the Assessment of Neuropsychological Status (RBANS) [15]	Average change (and variance) of scores over time in both treatment and control groups
Secondary – Efficacy	Demographics collected at baseline	Demographic composition of a study sample
Secondary – Efficacy	Intraclass correlation coefficient (ICC)	Similarity between treatment and control arm participant demographics
Secondary – Efficacy	Alzheimer's Disease Cooperative Study - Activities of Daily Living​ Inventory (ADCS-ADL) [16]	23-item assessment of activities of daily living
Secondary - Efficacy	Alzheimer's Disease Cooperative Study - Activities of Daily Living​ Prevention Instrument (17)	20-item assessment of activities of daily living for use in prevention trials
Secondary – Efficacy	Clinical Dementia Rating Scale (CDR) Scale [17]	A scale to characterize six domains of cognitive and functional performance
Secondary – Efficacy	Center for Epidemiologic Studies Depression Scale (CES-D) [18]	20-item assessment of symptoms of depression
Secondary – Efficacy and safety	Montreal Cognitive Assessment (MoCA) [19]	Screening assessment of mild cognitive impairment
Secondary – Efficacy	Imaging biomarker	Amyloid PET
Secondary – Safety	Eligibility screening	EKG and MRI without contrast
Secondary – Safety	Health status	Vital signs, physical examination, neurological examination, medical history, chemistry panel, urinalysis, concomitant medications, and adverse events

**Aim 1 analytic plan.** Percentages of participants who are a) eligible to participate but chose not to enroll and b) randomized but are lost to follow-up prior to protocol completion will be calculated along with 95% confidence intervals. Enrollment and loss to follow-up percentages will be compared between arms using Fisher's exact tests. Permutation tests will be used to assess if any baseline subject characteristics are associated with overall loss to follow-up percentages, or time to loss to follow-up.

**Aim 2 analytic plan.** Analysis of secondary endpoints in the total randomized population and by arm will be described using mean±standard deviation, or percentage, as appropriate and for summary measures 95% confidence intervals will be reported. For demographic characteristics, permutation tests, Fisher's exact test, or Mann-Whitney U-tests will be used. Absolute and relative change in RBANS from baseline to 24 months will be calculated using T-tests or Mann-Whitney *U* test. As the RBANS is also collected at 12 months, a random effects model will be used to assess the potential effect of each demographic variable on change in RBANS. Finally, interclass correlation (ICC) will be calculated by dividing the random effects variance in the random effects model (above) by the total variance.

## 3 Discussion

Dementia, specifically AD, is a growing concern within the aging population [2]. With evidence suggesting that disease progression starts well before symptoms appear [2], a prophylactic given before symptom onset holds potential to prevent AD. Memantine hydrochloride targets NMDA receptors that are functionally compromised by the coordinated action of toxic forms of Aβ and tau in a way that makes it a likely candidate for preventative treatment [10,12,20].

This feasibility trial has multiple strengths including a target drug which has shown clinical efficacy in moderate to severe AD. Memantine hydrochloride also has a favorable safety profile [20]. Additionally, the translational approach from basic science to randomized clinical trial has the potential to change the trajectory of people at risk for AD. The inclusion of a post-treatment amyloid PET scan is minimal risk while potentially providing valuable data about memantine's efficacy in a prevention context. Amyloid plaque burden is widely used as a biomarker for evaluating potential disease-modifying effects of Alzheimer's therapeutics, including recently approved agents such as monoclonal antibodies targeting amyloid. The small sample size, while a weakness in terms of evaluating efficacy and beyond the scope of this trial, is a strength of the trial in determining feasibility.

There are a few limitations for this design. The enrollment age of slightly before onset of AD symptoms may potentially limit our ability to see protection related to the drug. Similarly, the short duration (2 years) of the trial limits the potential to see effects of memantine on participants. While efficacy is beyond the scope of this trial, these limitations provide valuable insight on the secondary outcome of design features needed for a Phase III trial.

In summary, this paper presents the rationale, design, and protocol for the feasibility pilot trial of memantine hydrochloride vs. placebo to prevent the development of cognitive symptoms related to AD in at-risk adults. This feasibility trial focused on preliminary and exploratory outcomes needed to design a Phase III efficacy trial of memantine hydrochloride to prevent AD. We expect the trial will have acceptable retention rates and will provide significant direction on design elements needed for the Phase III trial.

## Funding

The Stanley E. Fulton Family Foundation and the University of Virginia Brain Institute.

## CRediT authorship contribution statement

**Anna M. Arp:** Data curation, Investigation, Writing – original draft. **George S. Bloom:** Conceptualization, Writing – review & editing. **Anelyssa D'Abreu:** Conceptualization, Investigation, Writing – review & editing. **Amy Fansler:** Methodology, Project administration. **Kimberlee Meegan:** Investigation. **Sarah Ratcliffe:** Methodology. **Jaideep Kapur:** Conceptualization, Funding acquisition, Methodology. **Carol Manning:** Conceptualization, Investigation, Methodology, Resources, Supervision, Writing – original draft.

## Declaration of competing interest

The authors declare that they have no known competing financial interests or personal relationships that could have appeared to influence the work reported in this paper.

## Data Availability

No data was used for the research described in the article.
